# Stellate Trichomes in *Dionaea muscipula* Ellis (Venus Flytrap) Traps, Structure and Functions

**DOI:** 10.3390/ijms24010553

**Published:** 2022-12-29

**Authors:** Bartosz J. Płachno, Małgorzata Kapusta, Piotr Stolarczyk, Piotr Świątek

**Affiliations:** 1 Department of Plant Cytology and Embryology, Institute of Botany, Faculty of Biology, Jagiellonian University in Kraków, 9 Gronostajowa St., 30-387 Kraków, Poland; 2 Department of Plant Cytology and Embryology, Faculty of Biology, University of Gdańsk, 59 Wita Stwosza St., 80-308 Gdańsk, Poland; 3 Department of Botany, Physiology and Plant Protection, Faculty of Biotechnology and Horticulture, University of Agriculture in Kraków, 29 Listopada 54 Ave., 31-425 Kraków, Poland; 4 Institute of Biology, Biotechnology and Environmental Protection, Faculty of Natural Sciences, University of Silesia in Katowice, 9 Bankowa St., 40-007 Katowice, Poland

**Keywords:** arabinogalactan proteins, carnivorous plants, cell wall, *Dionaea*, Droseraceae, hydathodes, transfer cells, wall ingrowths

## Abstract

The digestive organs of carnivorous plants have external (abaxial) glands and trichomes, which perform various functions. *Dionaea muscipula* Ellis (the Venus flytrap) is a model carnivorous plant species whose traps are covered by external trichomes. The aim of the study was to fill in the gap regarding the structure of the stellate outer trichomes and their immunocytochemistry and to determine whether these data support the suggestions of other authors about the roles of these trichomes. Light and electron microscopy was used to show the trichomes’ structure. Fluorescence microscopy was used to locate the carbohydrate epitopes that are associated with the major cell wall polysaccharides and glycoproteins. The endodermal cells and internal head cells of the trichomes were differentiated as transfer cells, and this supports the idea that stellate trichomes transport solutes and are not only tomentose-like trichomes. Trichome cells differ in the composition of their cell walls, e.g., the cell walls of the internal head cells are enriched with arabinogalactan proteins (AGPs). The cell walls of the outer head cells are poor in both low and highly homogalacturonans (HGs), but the immature trichomes are rich in the pectic polysaccharide (1–4)–β-D-galactan. In the immature traps, young stellate trichomes produce mucilage which may protect the trap surface, and in particular, the trap entrance. However, the role of these trichomes is different when the outer head cells collapse. In the internal head cells, a thick secondary wall cell was deposited, which together with the thick cell walls of the outer head cells played the role of a large apoplastic space. This may suggest that mature stellate trichomes might function as hydathodes, but this should be experimentally proven.

## 1. Introduction

Plant carnivory is largely a substitute for environmentally limited macroelements [1]. The traps of carnivorous plants, which digest prey, are analogous to the animal digestive tract [2]. Their digestive glands have attracted the attention of cytologists and are used as model systems, e.g., [3,4,5,6,7]. However, the digestive organs of carnivorous plants also have external (abaxial) glands and trichomes [8,9,10], which unfortunately have not been the focus of research. Depending on the type, they can fulfill different functions, e.g., providing protection (nonglandular trichomes—*Nepenthes*), secreting mucilage (*Aldrovanda*) or nectar (*Nepenthes*), and they can also play the role of hydathodes (*Cephalotus*, Sarraceniaceae, *Pinguicula*) [8,11]. Fineran and Lee [12,13] described the ultrastructure and ontogeny of external trichomes in *Utricularia* in detail. They proposed that the function of these external glands changes during ontogeny. At first, they are responsible for absorbing solutes from the external medium, but later, the mature external glands are responsible for secreting water from the trap.

In *Dionaea muscipula* Ellis (the Venus flytrap), the stellate outer trichomes (glands) are present on the entire outer trap surface and on the marginal teeth [9,10,14]. Dipalma et al. [14] suggested that these trichomes act as touch sensors or receptors, which eventually close off the trap, independently of the sensitive trigger hairs. However, Stanescu et al. [15] believe that these hairs play a protective role. According to Juniper et al. [10], in mature trichomes, the endodermoid cells appear to be highly active and accumulate chloride ions, while the outer head cells collapse. However, no ultrastructure data or cytochemical results were presented.

*D. muscipula* is an important model species especially when investigating the evolutionary “roots” of carnivory in plants, e.g., [16,17,18,19,20,21]. Its digestive glands have been extensively studied by various authors [5,6,22,23,24]. Therefore, we wanted to fill in the gap regarding the structure of the stellate outer trichomes (glands) and their immunocytochemistry and determine whether these data support the suggestions of other authors about the roles of these trichomes. Recently, we studied the carbohydrate epitopes that are associated with the major cell wall polysaccharides and the glycoproteins in the digestive glands of *Aldrovanda vesiculosa* and *D. muscipula* [24,25]. Therefore, the objective was to compare these digestive glands with the stellate trichomes, especially due to the fact that the transfer cells in the digestive glands were enriched in AGPs (which could be marker molecules for transfer cells).

## 2. Results

### 2.1. Trichome Structure

**Figure 1:**
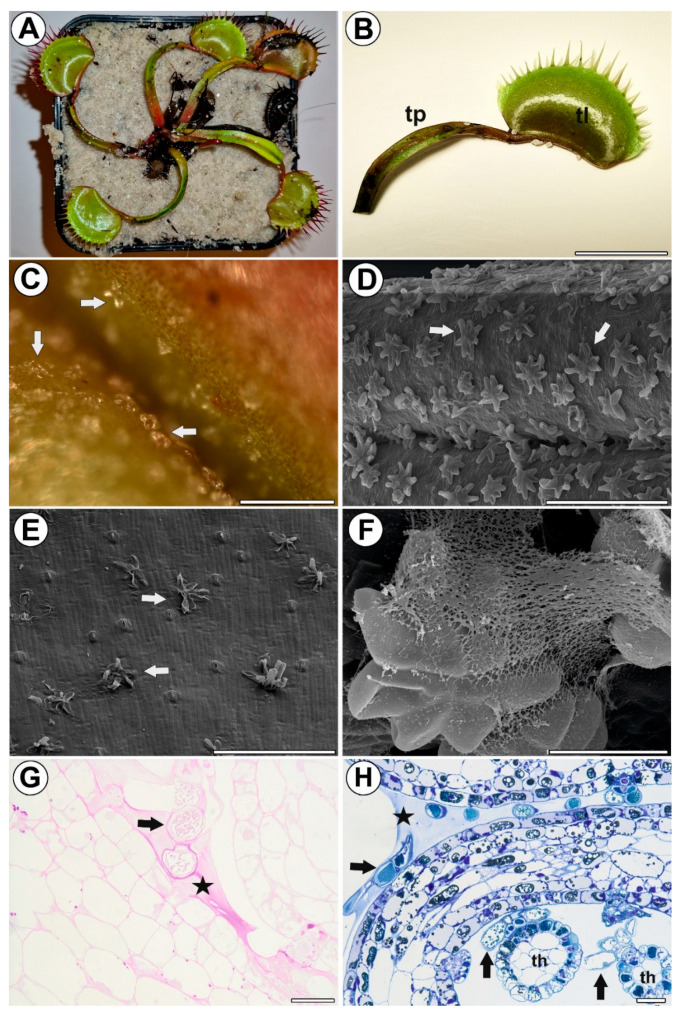
Distribution and morphology of the stellate trichomes of the *Dionaea muscipula* traps (**A**) *D. muscipula* plant. (**B**) Leaf morphology; trap petiole (tp), trap lobe (tl), bar 1 cm. (**C**) Stellate trichomes (arrow) on a young, non-opened trap surface, bar 250 µm. (**D**) Stellate trichomes (arrow) on a young, non-opened trap surface, bar 300 µm (scanning electron microscopy—SEM). (**E**) Stellate trichomes (arrow) on the external surface of a mature trap; note that that stellate trichomes have collapsed outer head cells, bar 300 µm (SEM). (**F**) Secretion on the surface of a stellate trichome from a young trap, bar 30 µm (SEM). (**G**) Positive result of the PAS reaction of the secretion between the trap lobes (star), stellate trichome (arrow), and bar 20 µm (light microscopy). (**H**) Section of a young, non-opened trap; note the secretion between the trap lobes (star) and stellate trichomes (arrow) on the marginal teeth (th), bar 25 µm.

Stellate trichomes were present on the trap petiole, the outer part of the trap lobes, and the marginal teeth, as well as at the peripheral band of the upper side of the trap lobes (Figure 1A–E). In the immature traps, some trichomes were covered by secretions (Figure 1G) (polysaccharide character—a positive result of the PAS reaction). The secretion also covered the space between the trap lobes in the immature traps (Figure 1H). Water droplets or secretions were not observed on the mature trichomes.

**Figure 2:**
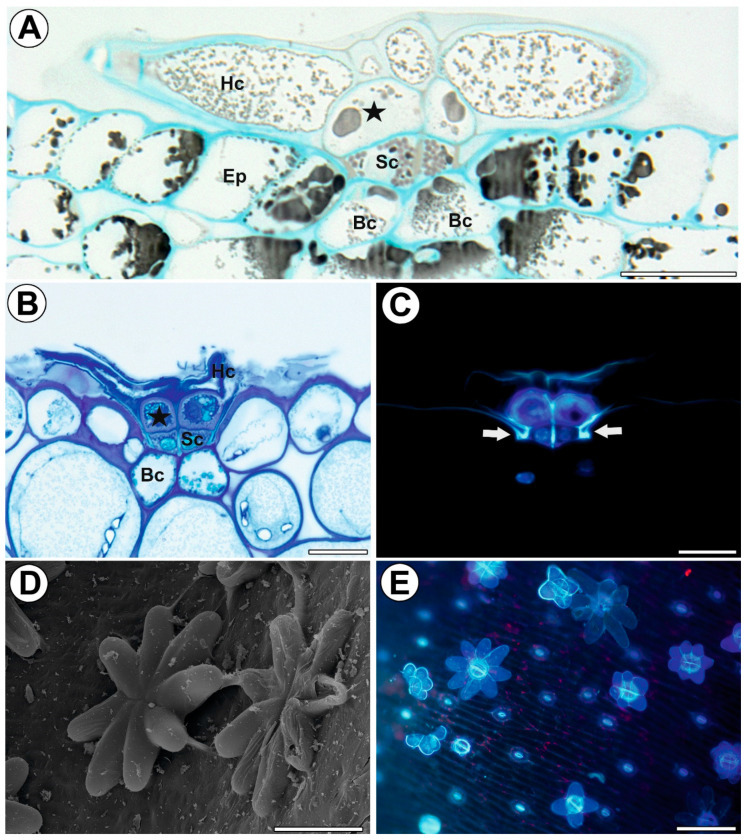
Structure of the stellate trichomes of the *Dionaea muscipula* traps. (**A**,**B**) A semi-thin section of a stellate trichome; outer head cell (Hc), internal head cell (star), stalk cell (Sc), basal cell (Bc) and an ordinary epidermal cell (Ep), bar 20 µm. (**C**) Section of a stellate trichome; autofluorescence of the cell walls, nuclei that had been treated with DAPI; note the strong autofluorescence of the cutinized cell walls (arrow), bar 20 µm. (**D**) Morphology of a stellate trichomes from a young trap, bar 50 µm. (**E**) Stellate trichomes with variable numbers of outer head cells from a young trap, bar 100 µm.

**Figure 3:**
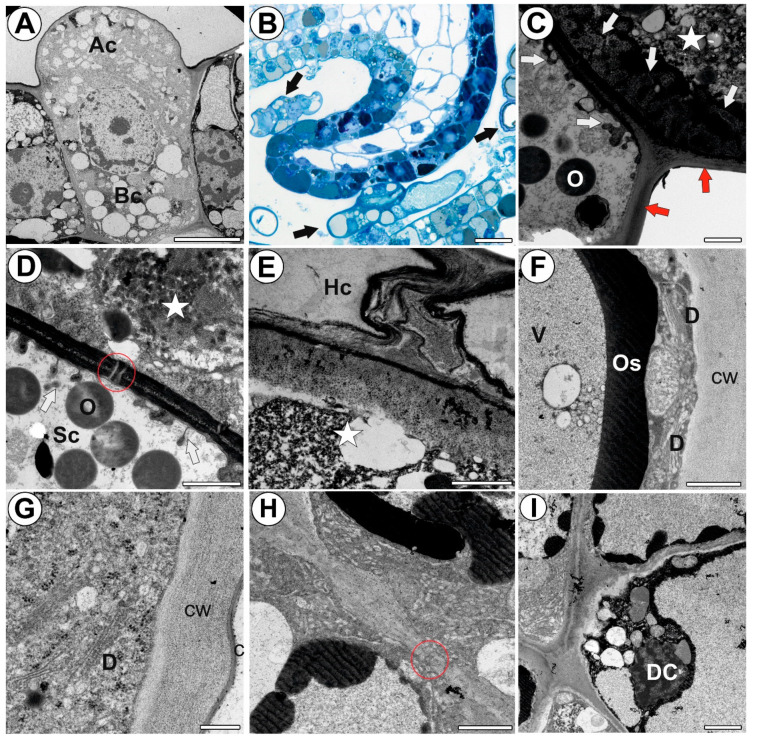
Development and structure of the stellate trichomes of the *Dionaea muscipula* traps. (**A**) The two-celled stage of a trichome, basal cell (Bc) and apical cell (Ac), bar 300 nm (transmission electron microscopy-TEM). (**B**) Various stages in the development of stellate trichomes (arrows), bar 20 µm (light microscopy). (**C**,**D**) Ultrastructure of a stalk cell (Sc) and internal head cell (star); cell wall ingrowths (arrows), plasmodesma (red circle), and oleosome (O), bars 1000 nm each (TEM). (**E**) Ultrastructure of an outer head cell (Hc) and internal head cell (star), bar 100 nm. (**F**,**G**) A part of a section of an outer head cell; dictyosomes (D), vacuole (V), osmiophilic material (Os), cuticle (c) and cell wall (cw), bars 800 nm and 400 nm, respectively (TEM). (**H**) Ultrastructure of outer head cells; note the plasmodesma (red circle), bar 900 nm, (TEM). (**I**) Outer head cells, note the degenerating outer head cells (DC), bar 2000 nm, (TEM).

Each stellate trichome consisted of two basal cells, two endodermal (endodermoid) stalk cells, and a head with two layers of cells (Figure 1A–C). The inner layer consisted of two cells (‘internal head cells’), while the outer layer consisted of up to eight elongated cells (‘outer head cells’), which were radially arranged and formed a star-shaped head (Figure 2D). The trichomes with four, six, seven, and ten external head cells were present next to each other (Figure 2E). The whole trichome was derived from a single epidermal cell via divisions. The epidermal cell became papillate and then divided into two daughter cells: basal and apical (Figure 3A). As a result of the next mitotic divisions, a trichome was formed (Figure 3B). During maturation, there were changes that were associated with the cell walls and the vacuolization of the trichome cells. The outer lateral walls of the stalk cells went through the cutinization process, and thus the Casparian strip was formed (Figure 3C). Moreover, the cell walls between the stalk cells were also cutinized (Figure 3C). The endodermal cells were differentiated into transfer cells. Cell wall ingrowths were formed at the transverse cell wall that neighbored the head cells (Figure 3C,D). The cytoplasm of the endodermal cells contained many oleosomes. The endodermal cells were connected to the head cells by plasmodesmata (Figure 3C,D). In the internal head cells, wall ingrowths were formed at the transverse cell wall that neighbored the endodermal cells. However, later, the next layer of wall material was deposited after which the ingrowths were buried under a layer of secondary wall material (Figure 3C). This thick layer of cell wall material was deposited on all of the cell walls (Figure 3E). In the mature trichomes, the head cells had thick outer walls. The mature outer head cells contained large vacuoles that contained osmiophilic, dark-stained deposits (Figure 3F). The vacuoles were surrounded by cytoplasm, which formed a thin layer underneath the outer peripheral cell walls where it mainly contained the elements of the ER, Golgi bodies, and small vesicles (Figure 3F,G). The head cells were covered by a cuticle (Figure 3G). The outer head cells were connected by plasmodesmata (Figure 3H). Some of these cells exhibited signs of degeneration, and there were cells that were still alive and degenerating cells even in the same trichome (Figure 2I). In the mature traps, the trichomes had collapsed outer head cells.

### 2.2. AGP Distribution

**Figure 4:**
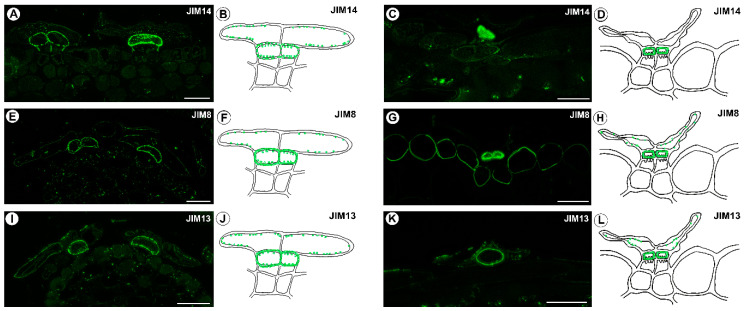
AGPs detected in the stellate trichomes of the *Dionaea muscipula* traps. (**A**) AGPs (labeled with JIM14) that were detected in the young trichomes, bar 20 µm. (**B**) Schematic occurrence (green) of the AGPs (labeled with JIM14) that were detected in a young trichome. (**C**) AGPs (labeled with JIM14) that were detected in a mature trichome, bar 20 µm. (**D**) Schematic occurrence (green) of the AGPs (labeled with JIM14) that were detected in a mature trichome. (**E**) AGPs (labeled with JIM8) that were detected in a young trichomes, bar 20 µm. (**F**) Schematic occurrence (green) of the AGPs (labeled with JIM18) that were detected in a young trichome. (**G**) AGPs (labeled with JIM8) that were detected in a mature trichome, bar 20 µm. (**H**) Schematic occurrence (green) of the AGPs (labeled with JIM8) that were detected in a mature trichome. (**I**) AGPs (labeled with JIM13) that were detected in a young trichomes, bar 20 µm. (**J**) Schematic occurrence (green color) of the AGPs (labeled with JIM13) that were detected in a young trichome. (**K**) AGPs (labeled with JIM13) that were detected in a mature trichome, bar 20 µm. (**L**) Schematic occurrence (green) of the AGPs (labeled with JIM13) that were detected in a mature trichome.

The epitope that is recognized by JIM14 mainly occurred in the cell walls of the inner head cells (intense signal) (Figure 4A). This AGP epitope was also observed as dots in the outer head cells (Figure 4A–D). However, this epitope was absent in the walls of the stalk (endodermoid) cells and the basal cells. The AGP epitope that is recognized by the JIM8 antibody was present in the outer head cells, but it was especially abundant in the cell walls of the internal head cells (Figure 4E–H). Although the AGP epitope that is recognized by JIM13 was mainly present in the cell walls of the internal head cells, it was also present in the outer head cells (Figure 4I–L).

### 2.3. Homogalacturonan Distribution

**Figure 5:**
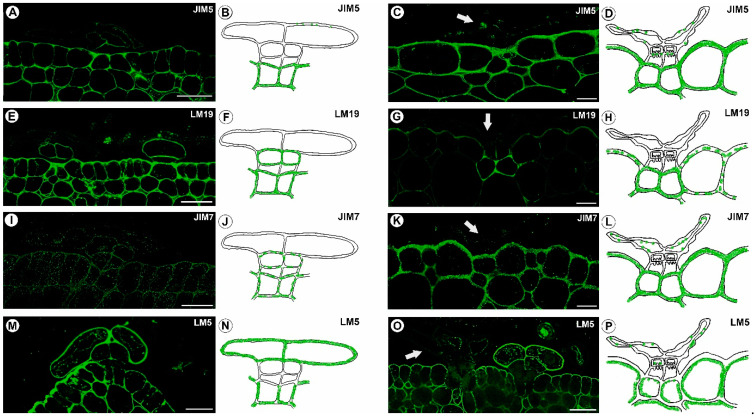
HGs that were detected in the stellate trichomes of the *Dionaea muscipula* traps. (**A**) HG (labeled with JIM5) that was detected in a young trichome, bar 20 µm. (**B**) Schematic occurrence (green) of the HG (labeled with JIM5) that was detected in a young trichome. (**C**) HG (labeled with JIM5) that was detected in a mature trichome (arrow), bar 20 µm. (**D**) Schematic occurrence (green) of the HG (labeled with JIM5) that was detected in a mature trichome. (**E**) HG (labeled with LM19) that was detected in the young trichomes, bar 20 µm. (**F**) Schematic occurrence (green) of the HG (labeled with LM19) that was detected in a young trichome. (**G**) HG (labeled with LM19) that was detected in a mature trichome (arrow), bar 20 µm. (**H**) Schematic occurrence (green) of the HG (labeled with LM19) that was detected in a mature trichome. (**I**) HG (labeled with JIM7) that was detected in a young trichome, bar 20 µm. (**J**) Schematic occurrence (green) of the HG (labeled with JIM7) that was detected in a young trichome. (**K**) HG (labeled with JIM7) that was detected in a mature trichome (arrow), bar 20 µm. (**L**) Schematic occurrence (green) of the HG (labeled with JIM7) that was detected in a mature trichome. (**M**) HG (labeled with LM5) that was detected in a young trichome, bar 20 µm. (**N**) Schematic occurrence (green) of the HG (labeled with LM5) that was detected in a young trichome. (**O**) HG (labeled with LM5) that was detected in a young trichome (on the right) and a mature trichome (on the left, arrow), bar 20 µm. (**P**) Schematic occurrence (green) of the HG (labeled with LM5) that was detected in a mature trichome.

A strong fluorescence signal that is detected by JIM5 (low methylesterified HGs) was observed in a trichome in the cell walls of the basal cells that are adjacent to the epidermal and parenchyma cells (Figure 5A–D). A weak signal or no signal was detected in the head cells (Figure 5A). The JIM5 epitope was detected within the walls of the epidermal and parenchyma cells of the traps. In a trichome, the fluorescence signal that is detected by LM19 (low methylesterified HGs) was observed in the cell walls of the basal cells, the stalk cells, and the internal head cells (Figure 5E,F). In the mature traps, these HGs occurred in the basal cells (Figure 5G,H). A fluorescence signal from highly esterified HGs (detected by JIM7) was observed in the cell walls of the basal cells, the stalk cells, and the internal head cells (Figure 5I–L). A delicate signal was observed in the cell walls of the outer head cells (Figure 5I–L). The JIM7 epitope was detected within the walls of the epidermal and parenchyma cells of the traps. In the immature trichomes, an intense signal from the pectic polysaccharide (1–4)-β-D-galactan (detected by LM5) was observed in the thick cell walls of the outer head cells (Figure 5M,N). A signal was also observed in the basal cells, but only a weak signal was recorded in the internal head cells (Figure 5O,P).

### 2.4. Hemicellulose Distribution

**Figure 6:**
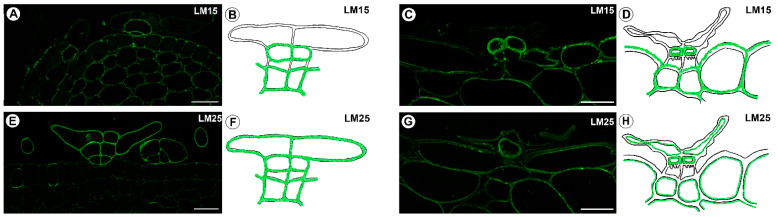
Xyloglucan that was detected in the stellate trichomes of the *Dionaea muscipula* traps. (**A**) Xyloglucan (labeled with LM15) that was detected in the young trichomes, bar 20 µm. (**B**) Schematic occurrence (green) of the xyloglucan (labeled with LM15) that was detected in a young trichome. (**C**) Xyloglucan (labeled with LM15) that was detected in a mature trichome, bar 20 µm. (**D**) Schematic occurrence (green) of the arabinogalactan proteins (labeled with LM15) that were detected in a mature trichome. (**E**) Xyloglucan (labeled with LM25) that was detected in the young trichomes, bar 20 µm. (**F**) Schematic occurrence (green) of the xyloglucan (labeled with LM25) that were detected in a young trichome. (**G**) Xyloglucan (labeled with LM25) that was detected in a mature trichome, bar 20 µm. (**H**) Schematic occurrence (green) of the xyloglucan (labeled with LM25) that was detected in a mature trichome.

A signal from xyloglucan (detected by LM15) was observed in the cell walls of the basal cells, the stalk cells and the internal head cells (Figure 6A–D). A very intense fluorescence signal from this xyloglucan was observed in the internal head cells in the mature traps (Figure 6C,D). The xyloglucan epitopes (detected by LM25) did occur in the cell walls in all of the cells of the trichomes in the immature traps (Figure 6E,F). There was a lack of these xyloglucan epitopes in the outer head cells in the trichomes from the mature traps, although they did occur in the internal head cells (Figure 6G,H).

### 2.5. Cell Viability Test

Most of the outer head cells of the young stellate trichomes that were analyzed were viable (79.16%, n = 48), and were visualized with fluoresceine diacetate only (Figure S1A) in contrast to the outer head cells of the mature trichomes, which were mainly non-viable (100%, n = 48), and stained with only propidium iodide without a fluorescein signal (Figure S1B).

## 3. Discussion

Our results regarding the general structure of the stellate trichomes are in agreement with the descriptions of trichomes by Juniper et al. [10]; however, we observed a larger number of outer head cells than those authors did. We found that the endodermal cells and internal head cells were differentiated as transfer cells, which was not recorded earlier for these trichomes. In the glands of carnivorous plants, the endodermal cells with wall ingrowths have been described in the tentacles in *Drosera* reviewed in [7,10], small trichomes in *Drosera* [10,26] but also in various trichomes in *Utricularia* [12,13,27,28,29] and in the digestive glands of *Aldrovanda vesiculosa* [24,30]. These wall ingrowths occur more commonly in the head cells of the glands in carnivorous plants of the genera: *Dionaea*, *Drosera*, *Drosophyllum,* and *Nepenthes* reviewed by [10]; *Pinguicula* [31]; *Aldrovanda* [24,30,32], and *Genlisea* [33]. According to Gunning and Pate [34], Offler et al. [35], and Offler and Patrick [36], transfer cells develop for the intensive short-distance transport between the symplast and apoplast. This supports the idea that the stellate trichomes transport solutes and are more than simply tomentose-like trichomes. It should be mentioned here that the occurrence of wall ingrowths is a common characteristic of both hydathodes and hydropotes [37,38,39]. However, since we did not observe active guttation, we could not confirm these functions here. We observed oleosomes in the endodermal cells in the stellate trichomes. Bemm et al. [40] observed oleosomes in the endodermal cells in the digestive glands of *D. muscipula*. These authors suggested that triacylglycerol from these organelles is a reservoir for the energy consumptive processes. We also suggest that triacylglycerol might be used for trichome cell activity.

In the immature traps, young stellate trichomes produce mucilage that might protect the trap surface, and, especially, the trap entrance (they protect both the immature digestive glands and the trigger hairs). We observed secretion that covered the trichomes as well as dictyosomes in the cytoplasm of the head cells. Thus, this might support the idea of Stanescu et al. [15] about its protective role, which has only been mentioned by these authors. We observed osmiophilic material in the vacuoles in the outer head cells; a similar material was observed in the digestive gland cells, which synthesize anthocyanins and naphthoquinones, of *D. muscipula* and other Droseraceae [41]. Synthesizing naphthoquinones makes plants less susceptible to herbivore infestations [42,43]. Therefore, young, developing traps will be “unsavory” for invertebrates; however, this should be experimentally tested in *D. muscipula*. According to Juniper et al. [10] the outer glandular cells collapse at the maturity of stellate trichomes. Our findings, both ultrastructural and viability test results, confirm the observations of these authors.

Juniper et al. [10] proposed that stellate trichomes closely resemble the external glands of the *Utricularia* traps due to the collapsing head cells and active endodermal cells. Finern and Lee [12,13] found that in the external glands of *Utricularia monanthos*, wall ingrowths were formed in the endodermal cells and also in the head cells. Although this is similar to *D. muscipula*, the difference is that in Venus flytraps, the ingrowths are absent in the outer head cells. In both species, a thick outer wall is formed in the head cells. Additionally, in both species, during trichome differentiation, the secondary wall material is deposited in the head cells (in *D. muscipula* in the internal head cells), and therefore the wall ingrowths becomes buried under the layers of secondary wall material (polysaccharide material). According to Fineran and Lee [12,13], such changes in the *U. monanthos* trichomes establish a standing osmotic gradient within the gland that provides the mechanism of secretion. Therefore, we think that the role of the stellate trichomes in *D. muscipula* is different after maturation because we observed that the outer head cells formed a large apoplastic space. Moreover, in the internal head cells, the thick secondary cell wall layer can function as an apoplastic space, which is necessary for the transport of water and ions. However, there is the question of whether these changes in the trichomes are associated with carnivory. In young, nonfunctional traps, both immature and fully differentiated stellate trichomes were present. One answer is that in mature traps, the stellate trichomes might help in the digestive cycle while another answer is that they simply help in water loss as typical hydathodes like in the plant species that grow in wet conditions, especially since Smith [44] observed stellate trichomes on the *D. muscipula* cotyledons. However, we could not confirm this function here, because we did not observe secretion on the mature trichomes. We think that an analysis of mutants with a nonfunctional or with no stellate trichomes would be helpful in solving this problem. We mainly observed AGPs in the cell walls of the inner head cells of the stellate trichomes, where cell wall ingrowths occurred and other secondary cell wall material was deposited. AGPs might provide positional information for the deposition of cell wall material [45] and they are connected with the formation of wall ingrowths [36,46]. Thus, our results are in agreement with the observations of the AGPs in the digestive glands in *A. vesiculosa* and *D. muscipula* [24,25], where AGPs were associated with the cell wall ingrowths. AGPs have been reported in the wall ingrowths in various plant species [47,48,49,50,51]. The presence of AGPs in the cell walls of the inner head cells of the mature stellate trichomes when the outer cells were dead supports both the important role of these cells and the activity of trichomes in mature traps. The types of stellate trichome cells differed in the composition of their cell walls, not only in the case of AGPs. The cell walls of the outer head cells were poor in both low and highly esterified HG but were rich in the pectic polysaccharide (1–4)-β-D-galactan in the immature trichomes. This galactan is usually deposited during plant cell expansion and differentiation, and it is involved in developing cell wall mechanical strength and elasticity [52,53,54], which would be consistent with our observations as the cells of the head undergo changes during development. When the stellate trichomes were fully differentiated, this galactan was lost in the thick walls of the outer terminal cells. When HGs were present in the cell walls, the basal cells of the trichomes were more similar to the epidermal and parenchyma cells than to the outer head cells of trichomes. Thus, the composition of the cell walls probably corresponds to a function of the type of trichome cells or to the mechanical role of their cell walls, especially because HGs are involved in many important characteristics of plant cell walls: porosity, elasticity, hydration, and also cellular adhesion/separation [55,56]. In both *D. muscipula* and *A. vesiculosa*, the cell walls of the parenchyma and the ordinary epidermal cells of the traps are rich in low and highly esterified HGs. It will be very interesting to compare the stellate trichomes with the digestive glands of *D. muscipula* regarding the occurrence of HGs and other polysaccharides.

Płachno et al. [24] after studying *A. vesiculosa* digestive glands, proposed that hemicelluloses should be present in the glandular structures of other species of carnivorous plants. The cell walls and cell wall ingrowths in the transfer cells of *A. vesiculosa* glands were rich in hemicelluloses: xyloglucan (LM15) and galactoxyloglucan (LM25). Similar to stellate trichomes, the cell walls of glandular cells and basal cells were rich in galactoxyloglucan (LM25). The differences concern the occurrence of xyloglucan (LM15) which is lacking in cell walls in the outer, terminal cells of *D. muscipula*. This difference may be due to the different ultrastructure of these cells related to the different roles of the trichomes in these species. However, it should be remembered that pectic homogalacturonan may mask abundant sets of xyloglucan epitopes in plant cell walls [54], which may affect the results presented.

## 4. Materials and Methods

### 4.1. Plant Material

The *D. muscipula* plants were purchased from a commercial supplier (GTP Harvey, Baniocha, Poland) and then cultivated at the Department of Plant Cytology and Embryology, Jagiellonian University in Kraków. The plants were grown in a mixture of sand and peat without fertilizer. For the trichome analysis, immature and mature traps were taken from mature plants at the same stage of development.

### 4.2. Histological and Immunochemical Analysis

The traps were fixed in 8% (*w*/*v*) paraformaldehyde (PFA, Sigma-Aldrich, Sigma-Aldrich Sp. z o.o. Poznań, Poland) and 0.25% (*v*/*v*) glutaraldehyde (GA, Sigma-Aldrich, Sigma-Aldrich Sp. z o.o. Poznań, Poland) in PIPES buffer overnight at 4 °C. The PIPES buffer contained 50 mM PIPES (piperazine-N,N′-bis [2-ethanesulfonic acid], Sigma-Aldrich, Sigma-Aldrich Sp. z o.o. Poznań, Poland), 10 mM EGTA (ethylene gly-col-bis[β-aminoethyl ether]N,N,N′,N′-tetraacetic acid, Sigma Aldrich, Poznań, Poland), and 1 mM MgCl_2_ (Sigma-Aldrich, Sigma-Aldrich Sp. z o.o. Poznań, Poland), pH 6.8. For the analysis of the occurrence of the major cell wall polysaccharides and glycoproteins, the plant material was embedded in LR White Resin (Polysciences Europe GmbH, Hirschberg an der Bergstrasse, Germany), which was repeated twice, and then sectioned. The rehydrated sections were blocked with 1% bovine serum albumin (BSA, Sigma-Aldrich) in a PBS buffer and incubated with the following primary antibodies—anti-AGP: JIM8, JIM13; JIM14 [57,58,59], anti-pectin: JIM5, JIM7, LM19, LM5 [57,60,61], and anti-hemicelluloses: LM25, LM15 [54,60,61,62] overnight at 4 °C. All of the primary antibodies were used in a 1:20 dilution. They were purchased from Plant Probes, UK, and the goat anti-rat secondary antibody conjugated with FITC was purchased from Abcam (Abcam plc, Cambridge, UK). The chromatin in the nuclei was stained with 7 µg/mL DAPI (Sigma-Aldrich, Sigma-Aldrich Sp. z o.o. Poznań, Poland) diluted in a PBS buffer and the samples were then cover-slipped using a Mowiol mounting medium: a mixture of Mowiol^®^4-88 (Sigma-Aldrich, Sigma-Aldrich Sp. z o.o. Poznań, Poland) and glycerol for fluorescence microscopy (Merck, Poland) with the addition of 2.5% DABCO (The Carl Roth GmbH + Co. KG, Germany). They were viewed using a Nikon Eclipse E800 microscope or a Leica DM6000B microscope. The photos were acquired as Z stacks and deconvolved using five iterations of a 3D nonblind algorithm (AutoQuant ™, Media Cybernetics Inc., Rockville, MD, USA). In order to maximize the spatial resolution, the images are presented as maximum projections. The stacks were obtained using a Leica DM6000B microscope equipped with a GFP filter. At least two different replications were performed for each of the analyzed traps, and about five to ten sections from each organ were analyzed for each antibody that was used. Negative controls were created by omitting the primary antibody step, which caused no fluorescence signal in any of the control frames for any of the stained slides (Figure S1C,D).

Semi-thin sections (0.9–1.0 µm thick) were prepared for LM and stained for the general histology using aqueous methylene blue/azure II (MB/AII) for 1–2 min [63]. The periodic acid–Schiff (PAS) reaction was also used to reveal the presence of any insoluble polysaccharides [64], and Sudan Black B (SBB) was used to detect the presence of any lipids or cuticle material [65]. Staining for total proteins was performed using mercuric bromophenol blue [66].

The traps were also examined using transmission electron microscopy (TEM) as follows. Fragments of the traps were fixed in a mixture of 2.5% glutaraldehyde with 2.5% formaldehyde in a 0.05 M cacodylate buffer (Sigma-Aldrich, Sigma-Aldrich Sp. z o.o. Poznań, Poland; pH 7.2) overnight or for several days, washed three times in a 0.1 M sodium cacodylate buffer, and post-fixed in a 1% osmium tetroxide solution at room temperature for 1.5 h (part of the material was fixed only in 1% osmium tetroxide solution in a 0.05 M cacodylate buffer—for rapid cell killing). This was followed by dehydration using a graded ethanol series and infiltration and embedding using an epoxy embedding medium kit (45359—Sigma-Aldrich). Following polymerization at 60 °C, sections were cut at 70 nm for the transmission electron microscopy (TEM) using a Leica ultracut UCT ultramicrotome, stained with uranyl acetate and lead citrate [67], and visualized using a Jeol JEM 100 SX microscope (JEOL, Tokyo, Japan) at 80 kV in the Department of Cell Biology and Imaging, Institute of Zoology, Jagiellonian University in Kraków, or a Hitachi UHR FE-SEM SU 8010 microscope at 25 kV, which is housed at the University of Silesia in Katowice.

### 4.3. Morphological Observations

For the SEM, the material was fixed and later processed as described in Lustofin et al. [68], and then dehydrated and dried using supercritical CO_2_. The material was then sputter-coated with gold and examined at an accelerating voltage of 20 kV using a Hitachi S-4700 scanning electron microscope, which is housed at the Institute of Geological Sciences, Jagiellonian University in Kraków, Poland.

The *D. muscipula* leaves were analyzed and photographed using a Nikon SMZ1500 stereoscopic microscope equipped with a digital DS-Fi1 camera (Precoptic, Warsaw, Poland).

### 4.4. Head Cell Viability Test

Sections of the traps (immature and mature) with young and mature stellate trichomes were immediately stained with a dual FDA/PI working solution. A fluorescein diacetate (FDA; Sigma-Aldrich, Sigma-Aldrich Sp. z o.o. Poznań, Poland) stock concentration of 1 g/mL in acetone and 2 μg/mL working solution of PI (Sigma-Aldrich, Sigma-Aldrich Sp. z o.o. Poznań, Poland) in a PBS buffer was used [69].

## 5. Conclusions

The occurrence of wall ingrowths in endodermoid and head cells supports the idea that stellate trichomes transport solutes and are more than simply tomentose-like trichomes.

The outer head cells collapse in mature trichomes.

In the internal head cells, a thick secondary wall cell was deposited, which together with the thick cell walls of the outer head cells played a role of a large apoplastic space

Trichome cells differ in the composition of their cell walls, e.g., the cell walls of the internal head cells were enriched with arabinogalactan proteins; however, the cell walls of the outer head cells were poor in both low and highly homogalacturonans.

Our cytological study indicates that stellate trichomes play different roles depending on their developmental stages. Young hairs secrete mucilage and then, after changes in the cells of the head, they change their function.

## Supplementary Materials

The following supporting information can be downloaded at: https://www.mdpi.com/article/10.3390/ijms24010553/s1.
