# Supplementary material for: Stellate Trichomes in Dionaea muscipula Ellis (Venus Flytrap) Traps, Structure and Functions
Source: Int J Mol Sci. 2022 Dec 29;24(1):553. doi: 10.3390/ijms24010553 (PMC9820793; doi:10.3390/ijms24010553)

Supplementary material Figure S1. The trichome head cell viability test and the Control reactions of the immunolabeling of the cell wall components that were detected in the trichomes. (**A**) Trichome head cell viability test in an immature trap, bar 20 μm. (**B**) Trichome head cell viability test in an immature trap, bar 20 μm. (**C**) Control reactions of the immunolabeling of the cell wall components that were detected in a young trichome, bar 20 μm. (**D**) A scheme of structure of the young trichome. (**E**) Control reactions of the immunolabeling of the cell wall components that were detected in a mature trichome, bar 20 μm. (**F**) A scheme of structure of the mature trichome.

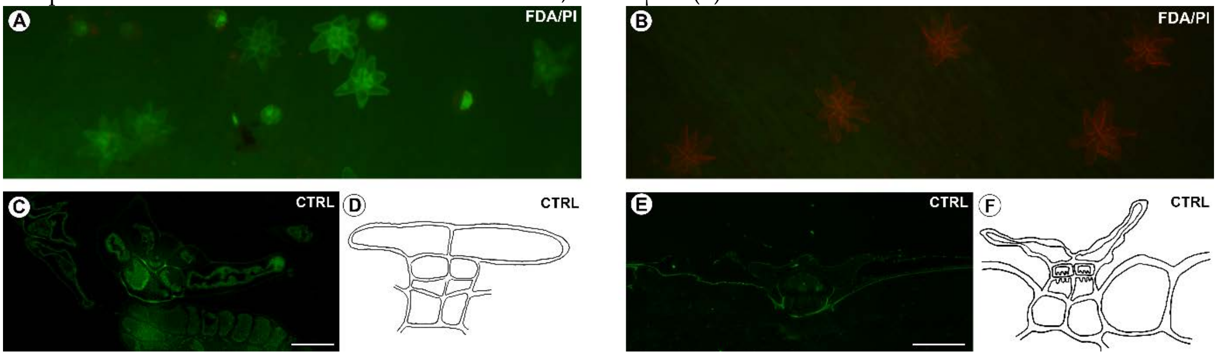

Supplement: Supplementary file 1 [file ijms-24-00553-s001.zip › ijms-2039158-supplementary.pdf]
